# Advertising

**Published:** 1914-11

**Authors:** 


### Advertising

You can buy for one cent five ounces of newspaper, printed and delivered. The paper unless in very large lots, costs $3\frac{1}{2}$ cents a pound wholesale. The mailing, delivery or newsboy's profit, the linotype work and printing, reporting, telegraph service, editing, maintenance of an elaborate plant and of expensive real estate are free, so far as you are concerned. You merely pay for the paper. With the exception of a few high priced magazines of general scope and some professional journals and transactions published on the co-operative plan and costing about double or three times what would be the price for comparable periodicals containing advertising, the same general principle applies to all periodical literature, although not usually to the same extent. For this reason, both as a matter of abstract justice, and, ultimately, for your own benefit, we ask you, so far as practicable, to favor advertisers in publications and to enable them to measure the value of such publicity by mentioning the periodical in which you saw their advertisement. Please note that we make this request not alone for this journal but for periodical literature generally.

As to this journal, we wish to report that considerable progress has been made toward the 50-page advertising section which, as announced some time ago, will warrant a reduction in the subscription to $1.50. This progress has been made in spite of continued hard times which, on account of the war will undoubtedly continue until we feel the benefit of the stimulation of home industries which the lack of imports will force upon us. The journal is in that tantalizing state in which a little more concentrated effort can vastly increase its ability to serve the interests of the profession of Western New York and increase, also, the number of readers who find it of value apart from any local interest. It can be stated with all sincerity that, as the journal has been financially able to do so, it has expanded and improved its contents. It is most desirable that it should expand somewhat further and improve still more. To do so, we ask the co-operation of all subscribers to extend its influence in a geographic sense, to secure the mutual support of professional organizations and the journal, to influence business firms with which they may have dealings or in which they are financially interested, to carry advertisements in the journal and to notify the editor of any firm, anywhere in western New York, which ought to advertise with us which does not.

